# Influence mechanisms of perceived organizational politics on the innovative behavior of newly hired university graduates

**DOI:** 10.3389/fpsyg.2026.1663534

**Published:** 2026-04-10

**Authors:** Junzhu Xu, Chongyun Wang, Jingmei Mo, Xiaodan Gao, Hu Li, Xinlong Li

**Affiliations:** 1 Sichuan University of Science and Engineering, Zigong, China; 2 Sichuan Key Provincial Research Base of Intelligent Tourism, Sichuan University of Science and Engineering, Yibin, China

**Keywords:** emotional intelligence, innovative behavior, new graduate employees, organizational identification, organizational justice, perceived organizational politics

## Abstract

With the deep implementation of an innovation-driven strategy, newly hired university graduate employees have become an important source of innovative vitality for enterprises, and the mechanisms for stimulating their innovative behavior warrant in-depth research. Based on a sample of 489 new graduate employees, this study employs structural equation modeling (SEM) to empirically test the impact of perceived organizational politics on innovative behavior and its underlying mechanisms. The results show that: (1) Perceived organizational politics directly inhibits the innovative behavior of new graduate employees, and also indirectly influences innovation through organizational justice and organizational identification. (2) Emotional intelligence plays a negative moderating role such that individuals with high emotional intelligence can effectively mitigate the adverse impact of perceived organizational politics on innovative behavior. These findings enrich the theoretical research on perceived organizational politics and employee innovation behavior, and provide practical insights for optimizing the management of new graduate employees and unleashing organizational innovation potential.

## 1 Introduction

Innovation has become a core driving force for firms to gain a competitive advantage and achieve sustainable development in today’s globally integrated knowledge economy (Porter and Kramer, 2011; Drucker, 2019). Among the many factors of innovation, employees with creative ability and motivation, often termed “innovative employees,” constitute one of the most fundamental and active elements in organizations (Zhou and Shalley, 2003). Existing research indicates that employees’ innovative behavior depends not only on individual traits (e.g., openness to experience and creative thinking) but also on the organizational environment (e.g., leadership style, team climate) and institutional support (e.g., incentive mechanisms, resource provision) (Amabile, 1998; Shafi et al., 2020). However, in practice, newly hired college graduate employees often encounter role ambiguity, a lack of social acceptance, and insufficient resource support during organizational entry. These challenges can stifle their innovation motivation and innovative behavior. In addition, cross-cultural studies show that differences in organizational culture (e.g., collectivist vs. individualist orientations across regions) can lead to differential impacts on new employees’ innovative behavior (Ashforth and Saks, 1996; Bauer et al., 2007). Therefore, probing the organizational contextual factors that influence the innovative behavior of new graduate employees is of significant theoretical and practical value (Kahn, 1990; Hofstede et al., 2004).

In recent years, with the innovation-driven development strategy being advanced, the innovative behavior of new graduate employees has become a rising topic in organizational behavior and human resource management research (Zhou and Hoever, 2014). As fresh blood in the firm, the innovation potential of new graduate hires holds special significance for organizational change and competitive advantage (Ferris et al., 2002). Perceived organizational politics, defined as an individual’s subjective perception of others’ self-serving behaviors in the organization, has been shown to negatively affect various employee attitudes, such as job satisfaction (Chesbrough, 2019). However, how such perceptions of organizational politics influence the innovative behavior of this particular cohort of new graduate employees remains under-researched. On one hand, new graduates carry systematic thinking and an exploratory spirit fostered by their academic training, and they possess frontier knowledge and technology that can inject innovative vitality into the firm. On the other hand, their innovative behavior is highly susceptible to the organizational socialization process (Perrot et al., 2014). This significant individual difference between them and senior employees may constitute a key contextual variable affecting their innovative performance, and the mediating pathway suggests a positive correlation between emotional intelligence and student social behavior. Although previous research has found that emotional intelligence is positively correlated with students’ social behavior, its role in helping new employees cope with organizational political contexts remains unclear. In particular, the influence mechanism of perceived organizational politics in this context remains to be explored in depth.

Based on these gaps, this paper aims to clarify the mechanism by which perceived organizational politics affects the innovative behavior of newly hired graduates (Goleman, 1998). To overcome the limitations of prior studies that often focused on a single pathway, we take a dual-path approach via organizational justice and organizational identification to examine the internal mechanism of the effect of perceived politics on innovation (Fredrickson, 2001). Furthermore, we investigate the moderating role of emotional intelligence to identify boundary conditions of this mechanism (Amabile, 1997). Specifically, we explore two core questions (Salovey and Mayer, 1990; Bandura, 1997; Diefendorff et al., 2005): (1) What is the internal logic by which perceived organizational politics influences the innovative behavior of new graduate employees, and do organizational justice and organizational identification mediate this relationship? (2) How does emotional intelligence moderate the effect of perceived politics on innovation, and what are the boundary conditions of this influence? Through empirical analysis, we aim to reveal how new graduate employees respond in terms of innovation when facing a politicized organizational context, and how individual emotional intelligence levels may strengthen or weaken these effects. Grounded in Conservation of Resources and Social Exchange theories, our conceptual model posits that perceived organizational politics impedes new employees’ innovative behavior by depleting their resources and undermining their sense of fairness.

## 2 Literature review and hypotheses

### 2.1 Perceived organizational politics and new graduate employees’ innovative behavior

Perceptions of organizational politics (POP) are defined as an individual’s subjective perception of the extent to which other members in the organization engage in self-serving, manipulative behaviors. Essentially, this concept captures an employee’s attribution that others in the organization pursue self-interest (the “self-serving” phenomenon), and it reflects the individual’s subjective appraisal of the work environment in terms of political behavior. As Ferris et al. (2002) elaborated, perceived organizational politics encompasses an individual’s cognitions of political behaviors in the organization, such as power plays and resource allocation, which together form one’s subjective judgment of the organizational political climate. When an individual perceives a strong political climate, their emotional attachment to the organization can be significantly affected. De Cuyper et al. (2012) further revealed the underlying mechanism of this influence: a strong perception of politics intensifies individuals’ psychological insecurity. A study of state-owned enterprise employees confirmed from the perspective of conservation of resources (COR) theory that this insecurity stems from employees’ fear that their own organizational resources (such as promotion opportunities and development space) will be harmed by political behaviors.

Compared to veteran staff, new graduate entrants experience even greater inner conflict when confronted with a highly political organizational climate. This cohort places a higher value on fairness, personal feelings, and career fulfillment, and they aspire to realize their self-worth in the organization. When they perceive organizational politics as “unfair,” the clash between their ideals and reality is intensified. According to COR theory, when individuals see organizational politics as a stressor, they tend to conserve their resources to avoid further loss. Kimura (2013) found that this resource conservation tendency reduces employees’ willingness to innovate. In real work settings, employees may fear that if an innovation attempt fails, their personal resources will be jeopardized, for example, investing substantial effort in a project only to receive no recognition due to political factors, and thus, they reduce innovative endeavors. Moreover, research suggests that the psychological stress and resource depletion triggered by perceptions of politics further undermine employees’ motivation and persistence to innovate. Many scholars agree that perceived organizational politics not only affects the initial willingness to engage in innovative behavior but also influences employees’ steadfastness when facing the pressures and setbacks inherent in innovation (Hou et al., 2018). A study focusing on new-generation employees (which includes many newly hired graduates) clearly showed that perceived organizational politics, through multiple pathways, weakens young employees’ innovative drive and their determination to persist in innovation.

Therefore, we propose:

H1: Perceived organizational politics is significantly negatively correlated with the innovative behavior of newly hired university graduate employees.

### 2.2 Mediating role of organizational justice

Organizational justice is typically divided into four dimensions: distributive justice, procedural justice, interpersonal justice, and informational justice. Distributive justice refers to the perceived fairness of outcome distributions related to organizational resources (including pay, benefits, honors, promotions, terminations, etc.) (Colquitt, 2001). Procedural justice denotes the perceived fairness of the processes by which organizational decisions are made (e.g., procedures for promotion, dismissal, performance appraisal) (Deutsch, 1975). Interpersonal justice concerns the degree of respect and propriety that authorities or superiors show when treating subordinates during the execution of procedures or communication of decisions (for example, whether they consider subordinates’ feelings or suggestions). Informational justice involves whether the individuals affected are provided with the necessary information and explanations, such as why certain procedures are used or why decisions are made in a particular way. Prior research has found a significant negative correlation between perceived politics and distributive justice (Leventhal, 1980; van Dick et al., 2004; Aggarwal et al., 2018). In other words, in highly political organizations, individuals with greater “influence” can use self-serving tactics that harm others’ interests, leading to outcomes based not on objective evaluation but on power, favoritism, or emotion, an allocation perceived as unjust. In such cases, employees feel their contributions are not proportional to rewards, and that incentives and promotions become uncertain, thus engendering strong feelings of unfairness.

Consequently, we propose:

H2: Perceived organizational politics is significantly negatively correlated with new graduate employees’ perceptions of distributive, procedural, interpersonal, and informational justice.

### 2.3 Organizational justice and organizational identification

Organizational identification refers to an individual’s sense of oneness with or belonging to an organization. It reflects the extent to which the individual psychologically accepts, integrates into, and feels attached to their organization (Tajfel, 1982). When an individual develops a strong organizational identification, they internalize the organization’s goals and values as their own and align their attitudes and behaviors closely with the organization. By identifying with a particular group or organization, individuals can fulfill important psychological needs such as enhancing self-esteem, achieving self-consistency, and obtaining self-fulfillment. From the perspective of the group engagement model, fair treatment in an organizational context is critical for the formation of organizational identification. When employees perceive fairness in key areas such as pay distribution, promotion opportunities, and decision-making participation, they are more likely to develop a strong sense of identification with the organization (Johnson et al., 2010; Hassan, 2013). This identification in turn drives them to exhibit behaviors beneficial to the organization, such as dedicating themselves to their work, proactively offering suggestions for organizational improvement, and staying committed and resilient even in difficult times.

Therefore, we propose:

H3: Organizational justice is significantly positively correlated with organizational identification.

### 2.4 Mediating role of organizational identification

Extant research has deeply explored the close links between organizational identification and employees’ attitudes and behaviors. In a meta-analytic review, Lee et al. (2015) found that organizational identification is significantly related to various employee attitudes and individual behaviors. When employees strongly identify with their organization, they become highly aligned with organizational goals and values. They are motivated to exert effort in exchange for rewards, whether material (e.g., performance bonuses) or psychological (e.g., supervisor recognition and praise), thereby fulfilling their sense of self-worth. Johnson et al. (2010) demonstrated that employees with high organizational identification have a much stronger motivation to engage in behaviors that promote superior organizational performance. Under pressures of market competition, such employees will proactively seek new market opportunities, actively experiment with optimizing work processes, and strive to improve efficiency, thus contributing to organizational performance under challenging conditions. Galvin et al. (2015), from a unique perspective, examined narcissistic organizational identification, a form of identification where individuals see themselves as central to the organization’s identity, which further enriched the research dimensions of organizational identification and employee relations. Kesen (2016) confirmed that organizational identification has a significantly positive effect on employees’ individual creativity. When employees identify with the organization, they tend to exhibit more creative behaviors.

Accordingly, we propose:

H4: Organizational identification is significantly positively correlated with the innovative behavior of newly hired university graduates.

### 2.5 Dual mediating effect of organizational justice and organizational identification

Prior studies on the impact of perceived organizational politics on employee innovative behavior have identified many critical mediating variables. For example, the perception of psychological contract breach has been found to mediate the relationship between politics and employee innovation behavior. Zhang and Ling (2013) showed through empirical analysis that job satisfaction serves as an important mediator influencing the relationship between politics and employees’ work attitudes. Lu et al. (2014) revealed a mediating mechanism involving psychological contract breach, in which diminished feelings of justice lead to lower organizational identification. Specifically, when employees do not feel fairly treated in the organization, observing unfairness in performance evaluations, pay distributions, and other key processes, their identification with the organization declines.

Combining our hypotheses H2–H4, we can clearly see the following chain for new graduate employees: higher perceptions of organizational politics lead to stronger feelings of unfairness in the organization, which in turn reduces perceptions of justice. This lack of justice further erodes their sense of identification with the company, and a lower organizational identification ultimately results in reduced innovative behavior. For instance, if a new hire frequently encounters unfair treatment due to organizational politics early in their tenure (e.g., feeling unfairly treated in project assignments or resource allocations), their identification with the firm will plummet. As a result, they will become far less willing to propose creative ideas or attempt new methods in their work, markedly diminishing their innovative behavior.

Therefore, we propose:

H5: Perceived organizational politics affects new graduate employees’ innovative behavior through the dual mediating effects of organizational justice and organizational identification.

### 2.6 Moderating role of emotional intelligence

Emotional intelligence (EI) is defined as an individual’s ability to monitor their own and others’ feelings and emotions, to discriminate among different emotions, and to use this information to guide one’s thinking and actions (Kaiafas, 2021). Individuals high in emotional intelligence possess acute emotional perception abilities; they can accurately detect subtle emotional changes in themselves and others and appropriately regulate emotions. Developing strong emotional intelligence helps individuals effectively control impulsive emotions and remain calm and optimistic under pressure, significantly enhancing their ability to cope with challenges.

On the one hand, new graduate employees (often part of the millennial generation) generally grew up in relatively stable and smooth environments, which can result in greater emotional volatility and poorer self-regulation when facing injustice, conflict, adversity, or stress. For example, when a newly hired graduate perceives politically driven unfairness in a project (such as inequitable resource allocation), they may lose emotional control, adversely affecting their work attitude and behavior. Indeed, Hou and Shao (2017) found that millennials’ emotional intelligence structure is such that those with lower emotional intelligence struggle more with emotional regulation in adverse situations. Emotional intelligence significantly affects how organizational politics are perceived by individuals and can indirectly influence employees’ attitudes and behaviors. From the perspective of behavioral motivation theory, employees with high emotional intelligence, when confronted with organizational politics, tend to attribute such political behaviors to internal factors (e.g., their own areas for improvement) rather than external injustice. This attribution style leads them to perceive a lower degree of organizational unfairness and, correspondingly, experience less negative motivational impact. For instance, in the face of seemingly unfair phenomena during a promotion competition, a high-EI employee will reflect on their own shortcomings rather than merely complain about external unfairness. Additionally, high-EI employees are more adept at leveraging their political skill, enabling them to deftly navigate and balance the negative feelings elicited by organizational politics (Sameer, 2018). Through effective communication and relationship management, they can garner both material and psychological support from colleagues and superiors, thereby replenishing positive motivational resources (Li et al., 2017; Hu and Luo, 2020).

Therefore, new graduate employees with high emotional intelligence can, through positive attribution and the exercise of political skill, reduce the adverse motivational effects caused by perceived politics, and may even gain additional drive to invest in innovation. In contrast, those with lower emotional intelligence, due to weaker emotion-regulation capabilities, are more prone to externalize blame and become dissatisfied with the organization when facing political behaviors, thus triggering negative work motivations. Lacking political savvy, they also find it difficult to offset the negative impacts brought by organizational politics. In practice, there are notable differences between new graduate hires and older employees in emotional management; these differences lead to distinct internal outcomes when perceiving organizational politics.

Accordingly, we propose Hypothesis 6, with sub-hypotheses for the direct and indirect pathways:

H6: Emotional intelligence plays a negative moderating role in the effect of perceived organizational politics on new graduates’ innovative behavior. In particular:

H6a1: Emotional intelligence negatively moderates the relationship between perceived organizational politics and organizational justice, such that the negative effect of politics on perceived justice is weaker when emotional intelligence is higher.

H6a2: Emotional intelligence negatively moderates the relationship between perceived organizational politics and organizational identification, such that the negative effect of politics on identification is weaker when emotional intelligence is higher.

H6b1: Emotional intelligence negatively moderates the indirect effect of perceived organizational politics on innovative behavior through organizational justice, i.e., the higher the emotional intelligence, the weaker the negative indirect effect of politics (via justice) on innovative behavior.

H6b2: Emotional intelligence negatively moderates the indirect effect of perceived organizational politics on innovative behavior through organizational identification, i.e., the higher the emotional intelligence, the weaker the negative indirect effect of politics (via identification) on innovative behavior.

In recent years, the academic community has developed diverse theoretical perspectives and research models concerning the mechanisms influencing employees’ innovative behavior. Previous studies, grounded in this theoretical foundation, have confirmed that situations such as unfair resource allocation and interpersonal conflicts triggered by perceptions of organizational politics can lead to the depletion of employees’ psychological capital and a decline in their motivation to innovate (Hou et al., 2022; Jiang, 2023). Regarding research on mediating mechanisms, existing models often focus on a single mediating pathway: some studies emphasize the mediating role of organizational justice, arguing that perceptions of organizational politics diminish employees’ willingness to innovate by undermining their sense of distributive and procedural justice (Aggarwal et al., 2018; Mustika, 2024); other research highlights the mediating effect of organizational identification, pointing out that the sense of alienation induced by organizational politics weakens employees’ sense of belonging to the organization, thereby reducing innovative behaviors beneficial to organizational development (Dick et al., 2019). In the exploration of boundary conditions, the moderating role of emotional intelligence has garnered widespread attention, with some studies treating emotional intelligence as an antecedent variable directly influencing innovative behavior (Hou et al., 2022; Ferdaws et al., 2020). In recent years, research on innovative behavior has exhibited characteristics of “multi-path integration” and “refinement of boundary conditions” (Tayyaba et al., 2020).

**FIGURE 1:**
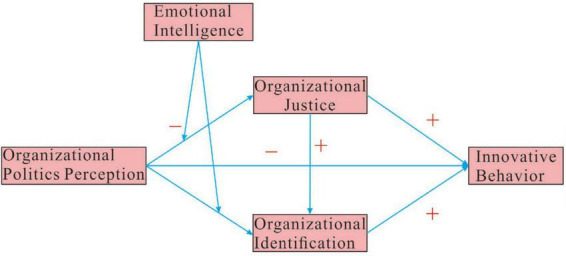
Research model.

In summary, grounded in Conservation of Resources Theory and Social Exchange Theory, this study will conduct the following research: Focusing on newly hired university graduate employees, it will examine the dual mediating effects of organizational justice and organizational identification in the relationship between perceived organizational politics and innovative behavior. Furthermore, it will test the moderating role of emotional intelligence in the impact of perceived organizational politics on organizational justice and organizational identification, as well as the resulting moderated mediation effects of the dual mediating pathways. This investigation aims to elucidate the boundary conditions of this influence mechanism, thereby providing theoretical support for constructing a more targeted cultivation model for the innovative behavior of new employees. The research model of this study is shown in Figure 1.

## 3 Methodology

### 3.1 Sample and procedure

This study employed a cross-sectional survey design, collecting data from newly hired employees via an online questionnaire in 2024. Grounded in China’s unique cultural context characterized by the interplay of “relationship-based interactions,” “collectivism,” and “intergenerational differences,” this study selected four provinces/municipalities for data collection: Beijing and Shanghai (eastern super-first-tier cities, comprising 31.2% and 28.7% of the sample, respectively) and Sichuan and Chongqing (key western provinces/municipality, comprising 25.4% and 14.7% of the sample, respectively). The survey, targeting employees hired within the past three years, was administered in October 2024 via the Credamo data collection platform. Anonymity and privacy protection measures were implemented to ensure data authenticity. A total of 525 questionnaires were distributed. After excluding invalid responses (e.g., those with over 10 consecutive identical answers or missing items), 489 valid questionnaires were retained, yielding an effective response rate of 93%. The sample demographics closely align with population characteristics within the Chinese cultural context across gender, age, education, and work tenure. Regarding gender, males constituted 66.7% (concentrated in male-dominated professions such as engineering and information technology), while females constituted 33.3% (predominantly in clerical, educational, and consulting roles). In terms of age, the “Post-95s” cohort (22–25 years old) comprised 62.3% (80.3% held bachelor’s degrees), the “Late-90s” cohort (over 25 years old) comprised 19% (82.4% had 1–3 years of work tenure), and those aged 22 and below comprised 18.7% (exhibiting weaker workplace adaptability). Educationally, master’s degree holders comprised 22.3% (often employed in research institutions and high-end manufacturing), while bachelor’s degree holders comprised 78.7% (distributed across producer services and traditional manufacturing). In work tenure, those with one year or less comprised 32.6% (accounting for 38.9% within the western sample), and those with 1–3 years comprised 67.4% (a higher proportion within the eastern sample).

Overall, the sample encompasses regional cultural disparities (eastern contractual spirit vs. western acquaintance society), gender role differentiation (traditional gender perceptions vs. gender-neutral trends), intergenerational value shifts (collective dedication vs. individual awakening), and educational stratification logics (research-oriented vs. application-oriented knowledge workers). This composition not only reflects the characteristics of China’s indigenous cultural dimensions but also establishes a robust foundation for the scientific validity and generalizability of the research findings.

### 3.2 Measures

We utilized well-established scales that have been widely used and validated in prior research to measure the main variables. All scales were rated on Likert-type scales. Following previous studies, we used a five-point Likert scale (1 = strongly disagree, 5 = strongly agree) for most measures unless otherwise noted. Higher scores indicate higher levels of the construct (All survey instruments were administered in Chinese; if a scale was originally in English, we used a standard translation-backtranslation procedure to ensure linguistic equivalence).

Innovative behavior (IB): We adopted the 6-item scale developed by Scott and Bruce (1994) to assess employee innovative behavior. This scale has been extensively used in prior studies and has demonstrated good validity. An example item is, “I often creatively propose original and practical solutions to work-related problems.” In this study, Cronbach’s α for the scale was 0.81.

Perceived organizational politics (POP): We used the 6-item scale developed by Hochwarter et al. (2003) to measure perceptions of organizational politics. A sample item is, “I feel that members of the organization spend too much time currying favor with those who can help them.” In this study, Cronbach’s α for this scale was 0.90.

Organizational justice (OJ): Perceptions of organizational justice were measured with four subscales: distributive justice, procedural justice, interpersonal justice, and informational justice. Distributive and procedural justice were measured using scales developed by Niehoff and Moorman (1993). The distributive justice subscale has 5 items, and the procedural justice subscale has 6 items, both rated on a 7-point scale (1 = strongly disagree, 7 = strongly agree). Interpersonal and informational justice were measured using scales developed by Colquitt (2001). The informational justice subscale has 5 items, and the interpersonal justice subscale has 4 items, also rated on a 7-point scale. In this study, the Cronbach’s α values for distributive, procedural, interpersonal, and informational justice were 0.79, 0.88, 0.90, and 0.92, respectively.

Organizational identification (OID): We measured organizational identification using the revised Mael’s Organizational Identification Questionnaire. This scale contains 6 items, rated on a 7-point Likert scale. Cronbach’s α for this scale in the present study was 0.82.

Emotional intelligence (EI): We adopted the 16-item scale developed by Law et al. (2004) to assess emotional intelligence. A sample item is, “I am quite sensitive to the feelings and emotions of others.” Cronbach’s α for this scale in our study was 0.87.

Control variables: Based on prior research, we controlled for several demographic variables that may influence innovative behavior: gender, age, education level, tenure in the current company, and team tenure (time working in the current team) were included as control variables in the analyses.

## 4 Results

### 4.1 Common method bias test and confirmatory factor analysis

We conducted both Harman’s single-factor test and a confirmatory factor analysis (CFA) to check for common method bias and to assess the discriminant validity of our measures. First, Harman’s one-factor test (exploratory factor analysis on all items of the five main constructs without rotation) showed that the first principal component accounted for 22.73% of variance, which is below the 50% threshold, suggesting that common method bias was not a serious concern in our data. Next, we performed CFA using Mplus 7.4 to evaluate discriminant validity among the five constructs (innovative behavior, perceived organizational politics, organizational justice, organizational identification, and emotional intelligence). As shown in Table 1, we compared our hypothesized five-factor model to four alternative models. The five-factor model yielded the best fit indices: χ^2^ = 509.954, χ^2^/df = 1.420, CFI = 0.928, TLI = 0.917, RMSEA = 0.047, all of which were significantly better than those of the alternative models. This confirms that the five key constructs have good discriminant validity.

**TABLE 1:** Confirmatory factor analysis results (*N* = 489).

Model	Factors (combined)	χ^2^	df	χ^2^/df	CFI	TLI	RMSEA
Five-factor model	IB/OP/OJ/OI/EI	509.954***	359	1.420	0.928	0.917	0.047
Four-factor model	IB/OP+OJ/OI/EI	756.518***	362	2.090	0.867	0.907	0.058
Three-factor model	IB/OP+OJ+OI/EI	945.327***	366	2.583	0.846	0.816	0.062
Two-factor model	IB+OP+OJ+OI/EI	1,137.215***	368	3.090	0.788	0.761	0.082
One-factor model	IB+OP+OJ+OI+EI	1,283.336***	371	3.459	0.639	0.613	0.104

IB, innovative behavior; POP, perceived organizational politics; OJ, organizational justice; OID, organizational identification; EI, emotional intelligence. “+” indicates factors combined into one.

***p < 0.001.

### 4.2 Descriptive statistics and correlations

We computed descriptive statistics and Pearson correlations for all major variables. The results are shown in Table 2. As expected, perceived organizational politics was significantly negatively correlated with innovative behavior (*r* = −0.342, *p* < 0.01), providing initial support for H1. Perceived organizational politics was also negatively correlated with organizational justice (*r* = −0.223, *p* < 0.01), supporting H2. Organizational justice was positively correlated with organizational identification (*r* = 0.305, *p* < 0.01), supporting H3. Furthermore, organizational identification was positively correlated with innovative behavior (*r* = 0.263, *p* < 0.01), supporting H4. These correlations align with our hypothesized relationships.

**TABLE 2:** Descriptive statistics and correlation matrix.

Variable	M	SD	1	2	3	4	5	6	7	8
Gender	1.389	0.476	0.237**	−0.007	−0.068	0.017**	−0.342**	−0.223**	0.305**	0.127**
Age	2.015	0.963								
Education	2.890	0.785	−0.105							
Company tenure	3.965	2.373	0.332**	0.648**						
IB	4.261	0.667	−0.039	−0.023	−0.105					
POP	3.986	0.995	−0.011	−0.079**	−0.033	0.037				
OJ	3.768	0.673	−0.051	−0.043	−0.038	−0.025	−0.042**			
OID	4.016	0.465	−0.045	−0.051	−0.036	−0.044	0.263**	−0.131**		
EI	4.138	0.529	−0.031	0.064	0.078	0.087	0.125**	0.107**	0.113**	

*N* = 489.

**Correlation is significant at the 0.01 level (two-tailed).

### 4.3 Dual mediation analysis of organizational justice and identification

**FIGURE 2:**
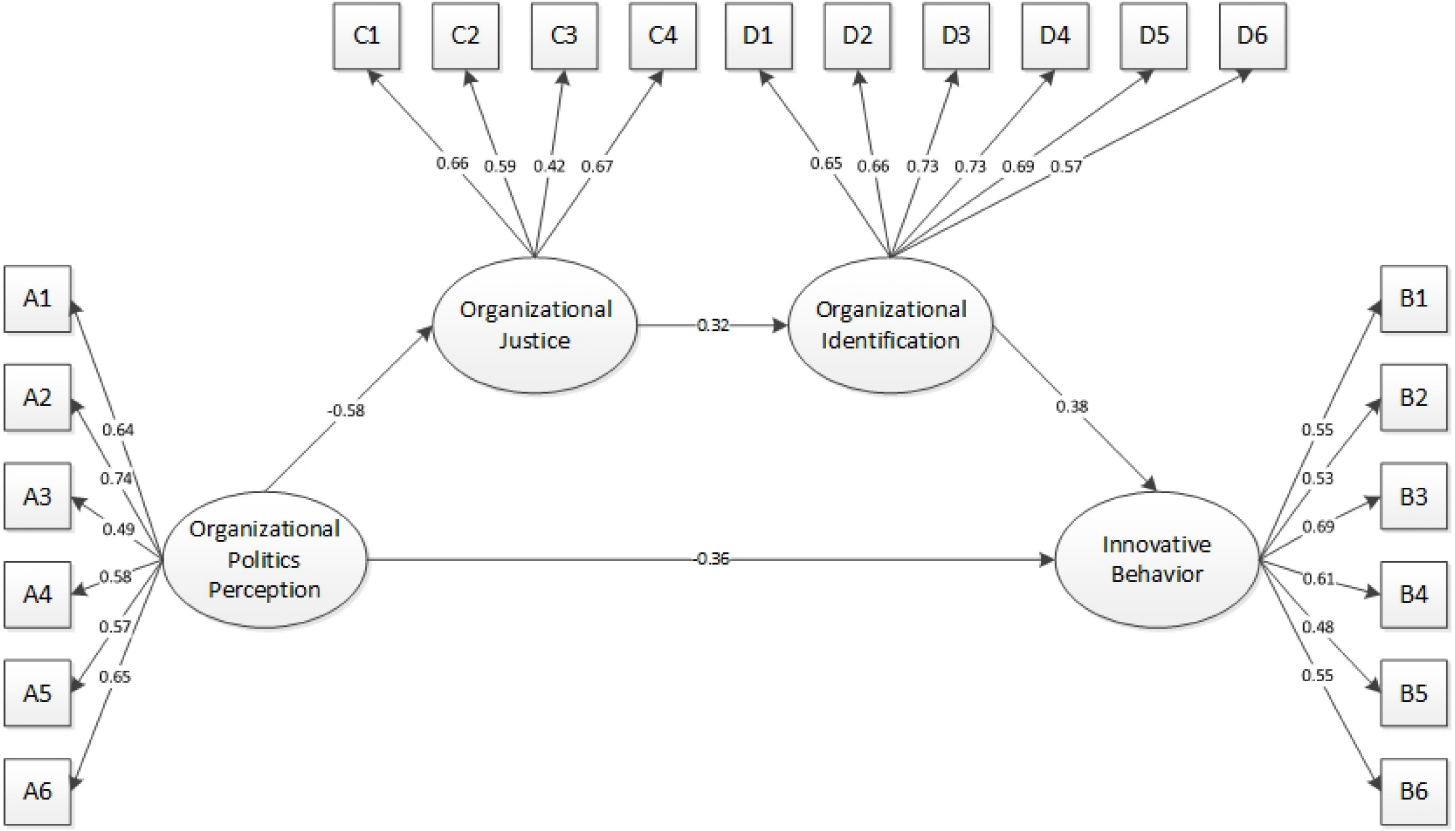
Organizational justice and organizational identification as dual mediators between perceived organizational politics and innovative behavior.

To test the dual mediating effect of organizational justice and organizational identification in the relationship between perceived politics and innovative behavior, we constructed a structural equation model (Figure 2) and conducted an SEM analysis using AMOS 26.0. The model fit indices indicate a good fit: χ^2^ = 432.473, df = 269, χ^2^/df = 1.608, CFI = 0.903, TLI = 0.899, RMSEA = 0.052.

All path coefficients in the model were statistically significant (*p* < 0.001). The standardized coefficients were as follows: perceived organizational politics → organizational justice = −0.58; perceived organizational politics → innovative behavior = −0.36; organizational justice → organizational identification = 0.32; organizational identification → innovative behavior = 0.38. Further analysis showed that the standardized indirect effect of perceived politics on innovative behavior (through organizational justice and identification) was 0.07. The direct effect (politics → innovation) was 0.36 in absolute value, and the total effect was 0.43. Thus, approximately 84% of the total effect is through the direct path and 16% through the mediating path. According to mediation analysis principles, perceived organizational politics not only influences innovative behavior directly, but also indirectly via organizational justice and identification. Through path analysis, we confirm that perceived politics exerts a dual mediating effect (via justice and identification) on new graduates’ innovative behavior. Therefore, H5 is supported.

### 4.4 Test of the moderating effect of emotional intelligence

We next examined the moderating effect of emotional intelligence on the relationships proposed in H6. We adopted a bootstrapping approach for the regression analyses, drawing 5,000 resamples with replacement and using 95% confidence intervals. The results are shown in Table 3. When organizational justice was the dependent variable, the interaction term between emotional intelligence and perceived politics was significant (β = −0.107, *p* = 0.031). This indicates a negative moderating effect of emotional intelligence on the relationship between politics and perceived justice, supporting H6a1. Likewise, when organizational identification was the dependent variable, the interaction of emotional intelligence and politics was also significant (β = −0.176, *p* = 0.004), indicating a negative moderating effect on the politics–identification relationship, supporting H6a2.

**TABLE 3:** Moderating effects of emotional intelligence on the impact of perceived organizational politics.

Variable	Organizational justice						Organizational identification					
	Coeff	SE	*T*	*P*	LLCI	ULCI	Coeff	SE	*T*	*P*	LLCI	ULCI
Gender	−0.033	−0.039	−0.798	0.376	−0.132	0.048	−0.069	0.039	−0.832	0.256	−0.157	0.035
Age	−0.018	0.053	−0.346	0.738	−0.088	0.065	−0.038	0.041	−0.428	0.690	−0.143	0.041
Education	−0.011	0.057	−0.172	0.862	−0.098	0.076	−0.014	0.036	−0.164	0.758	−0.086	0.079
Company tenure	0.069	0.046	1.875	0.047	−0.001	0.134	−0.028	0.035	1.659	0.051	−0.006	0.040
IB	0.095	0.162	0.558	0.753	−0.037	0.059	0.017	0.029	0.696	0.685	−0.046	0.062
POP	−0.075	0.026	−2.626	0.005	−0.175	−0.037	−0.061	0.027	−1.196	0.042	−0.126	−0.027
EI	0.562	0.062	7.904	0.001	0.467	0.594	0.455	0.058	8.067	0.001	0.259	0.573
EI*POP	−0.107	0.052	−2.265	0.031	−0.286	−0.016	−0.176	0.044	−2.371	0.004	−0.302	−0.034

**FIGURE 3:**
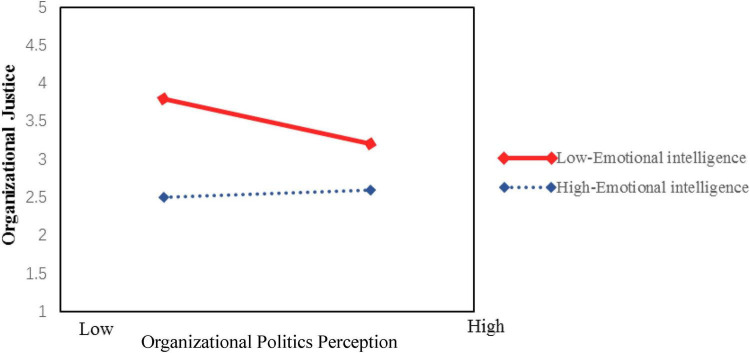
Moderating effect of emotional intelligence on the relationship between perceived organizational politics and organizational justice.

**FIGURE 4:**
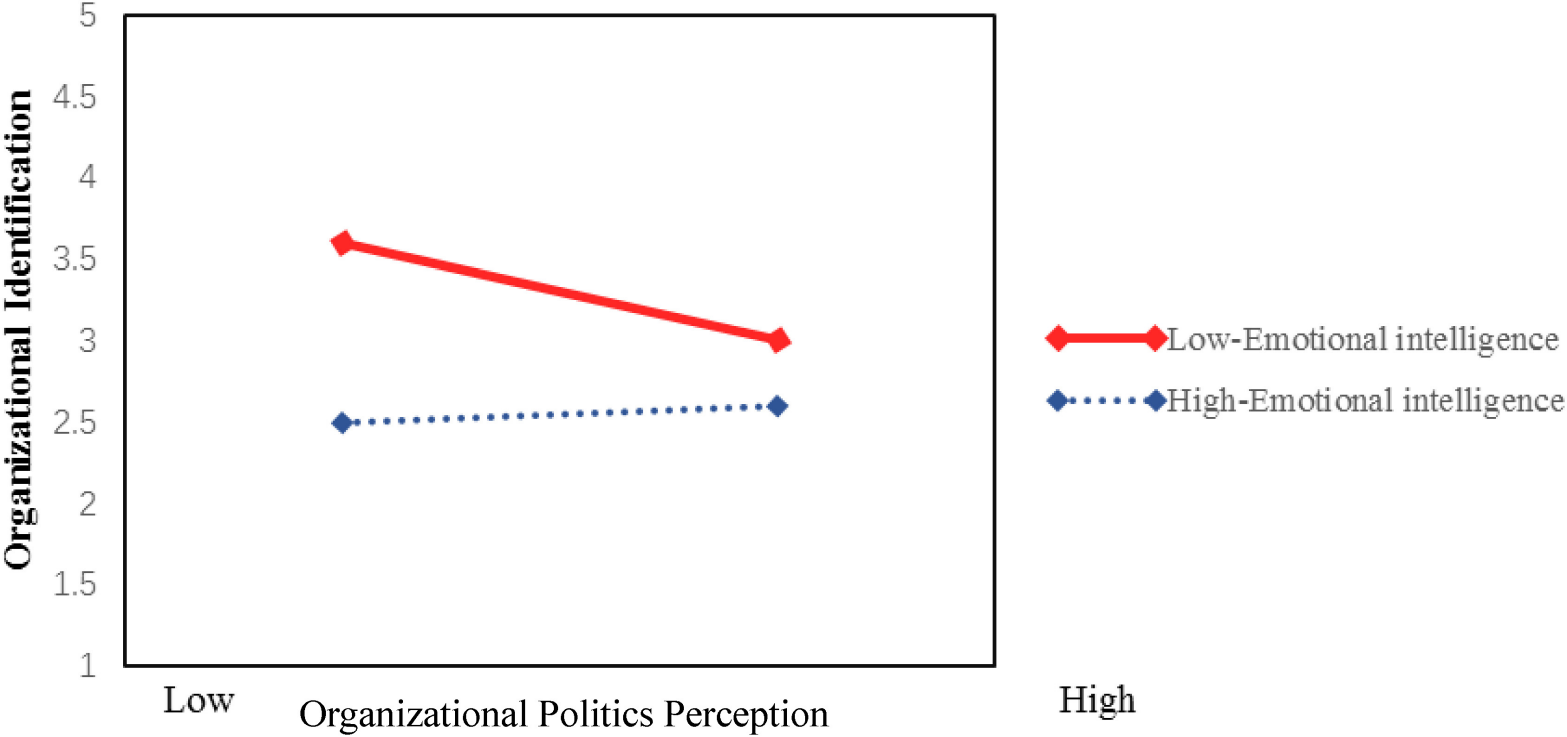
Moderating effect of emotional intelligence on the relationship between perceived organizational politics and organizational identification.

Further simple slope analysis revealed that, as shown in Figure 3, when newly hired graduates’ emotional intelligence was low (−1 SD), the negative effect of perceived organizational politics on organizational justice was not significant (β = −0.013, *p* > 0.05). However, when emotional intelligence was high (+1 SD), perceived organizational politics exerted a significant negative effect on organizational justice (β = −0.217, *p* < 0.001), thus supporting Hypothesis H6a1. Similarly, Figure 4 demonstrates a significant moderating pattern: under low emotional intelligence conditions, the impact of perceived organizational politics on organizational identification was non-significant (β = −0.036, *p* > 0.05), whereas under high emotional intelligence conditions, perceived organizational politics showed a significant negative effect on organizational identification (β = −0.309, *p* < 0.001), providing support for Hypothesis H6a2. We address this finding in the discussion.

### 4.5 Test of moderated mediation effects

Finally, we examined whether emotional intelligence moderates the indirect effects of perceived politics on innovative behavior via organizational justice and identification (H6b1 and H6b2). We again used a bootstrapping approach, testing conditional indirect effects at low (−1 SD) and high (+1 SD) levels of emotional intelligence and computing the index of moderated mediation for each pathway. The results are presented in Table 4.

**TABLE 4:** Results of moderated mediation effect tests (conditional indirect effects at ± 1 SD of EI).

Mediator	The indirect effect of regulation					The moderated mediating effect			
	Adjusting variable	Effect	BootSE	BootLLCI	BootULCI	INDEX	BootSE	BootLLCI	BootULCI
Organizational justice	Low/−1 SD	−0.006	0.0186	−0.048	0.031	−0.052	0.027	−0.106	−0.012
	High/+1 SD	−0.059	0.0190	−0.017	−0.035				
Organizational identification	Low/−1 SD	−0.009	0.0148	−0.008	0.029	−0.049	0.018	−0.074	−0.015
	High/−1 SD	−0.046	0.0155	−0.067	−0.019				

When emotional intelligence was low, the indirect effect of perceived politics on innovative behavior through organizational justice was β = −0.006, with a 95% bootstrap confidence interval of [−0.048, 0.031], which includes zero (indicating non-significance). In contrast, when emotional intelligence was high, the indirect effect via organizational justice was β = −0.059, with a 95% CI of [−0.067, −0.019], which does not include zero. Similarly, for the path through organizational identification, the indirect effect at low EI was β = −0.009 (95% CI [−0.008, 0.029], n.s.), but at high EI it was β = −0.046 (95% CI [−0.074, −0.015], significant). The index of moderated mediation for the justice-mediated path was −0.052 (95% CI [−0.106, −0.012]), and for the identification-mediated path was −0.049 (95% CI [−0.074, −0.015]), both of which have confidence intervals not including zero. These results indicate that the mediated effects of perceived politics on innovation via justice and via identification are each significantly moderated by emotional intelligence. In particular, the negative indirect effects are stronger (more pronounced) when emotional intelligence is higher. Thus, H6b1 and H6b2 are supported (though the direction of the moderation effect is opposite to what we theoretically anticipated, as we will discuss).

In summary, the overall model (Figure 1) is supported by the data: perceived organizational politics has a direct negative effect on new graduates’ innovative behavior (H1), as well as indirect negative effects via reduced organizational justice (H2 supported) and reduced organizational identification (H3 and H4 supported), confirming a dual mediation (H5). Emotional intelligence, however, unexpectedly exacerbated the negative effects of politics: high-EI individuals showed greater declines in perceived fairness and identification under high politics (opposite to the buffering we hypothesized), leading to stronger indirect negative effects on innovation (H6a1, H6a2, H6b1, H6b2 supported in terms of presence of moderation, though the direction is contrary to initial expectations).

## 5 Conclusion and implications

### 5.1 Key findings and discussion

Drawing on behavioral motivation theory, this study investigated the internal mechanism by which perceived organizational politics affects the innovative behavior of newly hired university graduates, with organizational justice and organizational identification as dual mediators and emotional intelligence as a moderator. The main findings are as follows:

(1) Perceived organizational politics significantly inhibits new graduate employees’ innovative behavior through the negative mediating effect of organizational justice. In theoretical terms, the presence of organizational politics often implies nonprocedural, nontransparent phenomena in areas like resource allocation and decision processes. New graduate employees, as fresh entrants, are in the early stages of accumulating psychological capital. Our findings confirm that perceptions of “procedural injustice” and “distributive injustice” triggered by organizational politics interact with characteristics common to this cohort—namely, emotional volatility, low psychological resilience, limited patience, and underdeveloped self-regulation. When they perceive a strong political atmosphere in the organization, their lack of experience in coping with such situations and limited adjustment abilities mean they often struggle to devise effective responses. This exacerbates the depletion of their psychological resources. The emotional reactions induced by perceived politics (such as frustration or anxiety) continuously drain their cognitive resources, dramatically reducing the subjective willingness they can devote to creative thinking. Ultimately, this produces a significant suppressing effect on their innovative behavior. This finding provides a new theoretical perspective for understanding the psychological mechanism through which organizational politics perceptions hinder individual innovation. Surprisingly, individuals with high emotional intelligence exhibited even stronger negative reactions under highly political conditions—possibly because their heightened sensitivity to injustice leads to greater psychological resource loss, which in turn further suppresses their innovative behavior.

(2) Perceived organizational politics further weakens new graduate employees’ innovative behavior by reducing their investment in the innovation process. Innovation process investment (the effort devoted to generating, promoting, and implementing new ideas) represents a dynamic psychological resource that is highly fragile under a political organizational context. Studies have shown that when employees perceive a strong organizational political climate, their innovation inputs tend to systematically decrease or even cease altogether. From a resource conservation standpoint, perceived politics triggers a continuous drain of employees’ emotional resources. New graduate hires, with relatively weak adjustment capacity, instinctively adopt resource-protection strategies in response to such drain. Given that innovative activities are high in uncertainty and resource consumption, these employees, to secure their footing in the organization, prioritize completing routine tasks and cut back on discretionary innovation efforts. Furthermore, based on expectancy-value theory, if organizational resource allocation is perceived to deviate from a performance orientation (e.g., rewards are not based on merit but on favoritism), such negative expectations drastically dampen their enthusiasm and intrinsic motivation for innovation. Because new graduate employees are in a period of career adjustment and are extremely sensitive to organizational fairness, while lacking the political skills to navigate complex interpersonal situations, once they detect organizational politics, the internal shock is profound. Compared to older employees, they are more easily discouraged by perceptions of politics, leading to a sharper reduction in innovation investment. This finding provides an important supplement to our understanding of factors influencing the innovative behavior of the new generation workforce, and it can help companies formulate management strategies to stimulate the innovation potential of new hires.

(3) The mediating role of psychological capital in the relationship between politics and innovation is significantly moderated by individual emotional intelligence. Although psychological capital was not directly measured in our study, our theoretical framing and results suggest that emotional intelligence conditions how perceived politics translates into loss of psychological resources and subsequent innovation outcomes. Newly hired graduate employees with high emotional intelligence can effectively buffer the negative impact of perceived organizational politics on psychological capital, thereby maintaining their innovative behavior. Immersed in a highly political organizational environment, these individuals are keenly aware of their own emotional fluctuations and can deploy adaptive strategies like cognitive reappraisal and stress management to cope with the uncertainties of organizational politics. By doing so, they reduce unnecessary emotional resource depletion and prevent their psychological capital (self-confidence, resilience, optimism, and hope) from deteriorating due to the accumulation of negative emotions. At the same time, high-EI employees are adept at finding growth opportunities in challenging situations, viewing organizational politics as a manageable reality of working life rather than as a purely threatening force. This outlook helps them sustain a high sense of self-efficacy and enhances their willingness to take risks in innovation. Thus, even when political perceptions are strong, they can keep their psychological capital stable and proactively seek external resources (such as mentor support or cross-department collaboration) to drive innovation. In stark contrast, low-EI employees, due to insufficient emotional regulation capability, are more likely to fall into anxiety or negative attribution cycles when perceiving organizational politics, which accelerates the exhaustion of their psychological capital and suppresses their innovative behavior. This finding extends the application of conservation of resources theory in the context of organizational politics by highlighting the role of personal emotional competencies.

(4) By constructing a moderated mediation model, this study reveals the mechanism and boundary conditions of how perceived organizational politics affects the innovation behavior of new-generation employees. The findings reveal that when employees perceive an organizational political climate, they tend to reduce their energy investment in the innovation process—encompassing idea generation, promotion, and implementation stages—as a resource conservation strategy, thereby inhibiting innovative behavior. Employees with high emotional intelligence possess precise attributional capabilities, enabling them to clearly identify that unfair phenomena in the organizational environment stem from external political behaviors rather than their own lack of competence (Qin and Yang, 2010). This acute insight prevents them from rationalizing injustice; instead, it elicits a stronger rejection of behaviors that violate their ethical standards (Galit and Eran, 2014). Concurrently, by utilizing their emotional regulation and resource acquisition abilities, they can effectively decouple “environmental problems” from “personal capability.” Given that self-efficacy is a core belief driving work performance (Stajkovic and Luthans, 1998), protecting this key personal resource is crucial for them. Therefore, in this context, emotional intelligence does not serve its conventional buffering role but rather amplifies the individual’s perception of the ethical costs and resource depletion inherent in organizational politics, ultimately manifesting as significantly reduced perceptions of organizational justice and identification. Meanwhile, this negative impact is moderated by emotional intelligence. Employees with high emotional intelligence possess a buffering mechanism that can break the “perceived politics–innovation suppression” cycle. Leveraging this emotional regulation capacity, individuals can maintain or even increase their innovation involvement despite psychological resource depletion, thereby sustaining stronger persistence in innovative behavior. This model extends the application of the Conservation of Resources Theory in innovation research and offers actionable intervention insights for management practice, such as implementing emotional intelligence development programs to enhance new-generation employees’ resilience toward organizational politics.

(5) Drawing on Hofstede’s cultural dimensions theory (Hofstede et al., 2004), the pronounced power distance within organizations compels newly hired graduates to suppress dissatisfaction despite perceiving organizational political behavior, adhering to “obedience consciousness.” This surface-level compliance internalizes into deeper perceptions of injustice, thereby amplifying the negative effect of perceived organizational politics (POP) on distributive, procedural, interpersonal, and informational justice. For instance, when confronted with power-dominated unfair resource allocation, employees readily develop a “uselessness of effort” mindset, which diminishes justice perceptions and exacerbates the suppression of innovative behavior—a finding consistent with studies on state-owned enterprise employees (Qin and Yang, 2010).

Concurrently, collectivist culture strengthens the mediating effect of organizational identification. As Tajfel’s (1982) social identity theory notes, individuals in collectivist settings place greater emphasis on psychological connections with their organization. New graduate employees tend to internalize organizational goals as their own; when perceiving organizational politics, those with stronger organizational identification are more inclined to adjust their behavior from a “collective interest maintenance” perspective, reducing innovation withdrawal induced by political perceptions. Moreover, the interpersonal collaboration emphasized by collectivism can enhance the positive influence of organizational identification on innovative behavior, such as seeking support through teamwork to sustain innovation enthusiasm. This aligns with van Dick et al.’s (2004) research on organizational identification and collective-oriented behavior. Together, these mechanisms constitute the core cultural moderating pathways of the model within the Chinese context.

### 5.2 Theoretical contributions

This study uncovers the influence mechanism of perceived organizational politics on the innovative behavior of newly hired university graduates, making several contributions to the literature:

(1) Contextualizing organizational politics research in China: We deepen the understanding of how perceived organizational politics affects employee innovation behavior in the Chinese context. The complexity of organizational politics is amplified in China’s guanxi (relationship-centric) organizational environment, yet existing research has mostly focused on how related skills or performance appraisal systems impact innovation, with relatively little attention on the internal mechanism of employees’ perceptions of politics. Our research provides a new perspective for understanding the innovative behavior of new graduate employees under the cultural backdrop of China, enriching the literature on organizational politics and innovation in a non-Western context.

(2) A dual-path model based on COR theory (state-path and process-path): Differing from prior studies that often examined a single pathway, our study—grounded in conservation of resources theory—constructs a dual-path model encompassing both a “psychological state” route and a “process” route. We reveal that organizational justice and organizational identification serve as mediators between perceived politics and innovative behavior, elucidating the internal mechanism by which the loss of psychological resources (due to perceived injustice) impacts innovation outcomes. This dual-path approach provides a more comprehensive explanation of how organizational politics can deplete employees’ resources (e.g., sense of fairness, sense of belonging) and thereby hinder innovative behavior.

(3) Extending COR theory by incorporating emotional intelligence as a moderator: By introducing emotional intelligence as a moderating variable, we expand the application of COR theory in the context of organizational politics and innovation. Existing research on innovation has mostly focused on organizational contextual factors (such as innovation support climate or differential leadership) and has neglected the role of individual capabilities. By accounting for new employees’ emotional management characteristics, our study finds that emotional intelligence can significantly alter the impact of perceived politics. Specifically, high-EI individuals are able to maintain their psychological resources through emotional regulation and garner interpersonal support, thereby weakening the negative effects of politics on psychological capital and innovation process investment, and attenuating the mediated impact of politics on innovation. This highlights the importance of personal resources and capabilities in conjunction with environmental factors, offering a nuanced view that merges individual differences with COR dynamics.

(4) In terms of managerial practice, our study suggests that organizations should cultivate a fair and transparent climate and provide emotional intelligence training or support for newly hired graduates to mitigate the inhibitory effects of organizational politics on their innovation.

### 5.3 Limitations and future research

Despite the theoretical and practical value of our findings, there are several limitations that should be acknowledged, which also point to directions for future research.

First, in terms of research design, our data relied on self-reports from new graduate employees, which raises the risk of common method bias. Although we took steps to test and control for this (e.g., anonymity, Harman’s test, CFA), the subjective nature of survey responses means that social desirability bias or memory bias may still influence the results to some extent. Future studies should consider collecting data from multiple sources, such as supervisor ratings or peer evaluations, to enhance data reliability.

Second, regarding levels of analysis, this study examined organizational politics solely from the individual perception perspective and did not incorporate the organizational-level political climate or culture. This single-level analysis constrains our understanding of the broader mechanism of organizational politics. Follow-up research could employ a cross-level design, simultaneously gathering data on organizational-level political atmosphere and individual-level perceptions, to more fully unveil the multi-level effects of organizational politics.

Finally, in terms of research scope, we primarily focused on the moderating role of emotional intelligence and did not include other potential boundary conditions that may influence the politics–innovation relationship. Future research could explore additional individual traits (e.g., core self-evaluation), job characteristics (e.g., job autonomy), and team or organizational factors (e.g., team innovation climate) as moderators to refine the theoretical model. Incorporating these factors would provide a more complete picture of when and for whom perceived politics most strongly affects innovation, thereby improving the model’s robustness.

Overall, despite these limitations, our study lays a theoretical foundation and offers practical insights for subsequent systematic research on the relationship between new-generation employees’ perceptions of organizational politics and their innovative behavior. We believe it holds important value for extending this line of research and informing management practice.

## Data Availability

The original contributions presented in this study are included in this article/supplementary material, further inquiries can be directed to the corresponding authors.
